# Do Locus of Control, Self-esteem, Hope and Shame Mediate the Relationship Between Financial Hardship and Mental Health?

**DOI:** 10.1007/s10597-019-00467-9

**Published:** 2019-09-24

**Authors:** Charlotte Frankham, Thomas Richardson, Nick Maguire

**Affiliations:** 1 School of Psychology, University of Southampton, Southampton, SO17 1BJ UK; 2 Mental Health Recovery Teams, Solent NHS Trust, St. Mary’s Community Health Campus, Milton Road, Portsmouth, POE 6AD UK; 3 Present Address: Ealing Early Intervention Service, West London NHS Trust, Cherington House, Cherington Road, Hanwell, W7 3HL UK

**Keywords:** Depression, Anxiety, Financial hardship, Mental health, Shame, Hope

## Abstract

In a longitudinal study of 104 participants, the psychological factors of economic locus of control, self-esteem, hope and shame were explored for their impact on the relationship between financial hardship and mental health. Participants completed measures of financial hardship, the psychological factors and measures of mental health three times at three-monthly intervals. A hierarchical regression analyses indicated that subjective financial hardship, hope and shame significantly predicted mental health outcomes. Mediation analyses demonstrated that hope mediated the relationship between subjective financial hardship and depression, stress and wellbeing; that shame mediated the relationship between subjective financial hardship and anxiety; and that neither shame nor hope mediated the relationship between subjective financial hardship and suicide ideation.

## Introduction

Mental health disorders have been consistently shown to have greater prevalence in lower Socio-Economic Status (SES) groups (Fryers et al. 2004). More specifically, rates of depression (Lorant et al. 2003), schizophrenia (Harrison et al. 2001) and admission to psychiatric hospital (Koppel and McGuffin 1999) are increased. The interaction between mental health and poverty is complex and influenced by a multitude of factors. Social drift proposes that downward social mobility as a consequence of mental health problems is at the root of these figures (Timms 1998) whereas social causation theory holds that poverty leads to emotional disturbance (Langner and Michael 1963).

Financial hardship describes situations in which individuals have insufficient economic resources required to sustain a home, pay bills and debts, and meet essential costs, such as food and transportation (Mirowsky and Ross 2001). Measures of financial hardship ascertain the severity of deprivation by establishing the extent to which essential costs are being met (Mack and Lansley 1985), and may therefore be a more reliable indicator of the relationship between financial disadvantage and mental health (Fryers et al. 2003). Going without meals, seeking assistance from community organisations, and having to pawn or sell possessions have all been associated with depression (Butterworth et al. 2012), just as deteriorations in mental health have been associated with the inability to meet housing costs (Mason et al. 2013) or heat the home (Butterworth et al. 2009).

Financial hardship places individuals at an increased risk of developing mental health problems (Kiely et al. 2015). Indeed research suggests that hardship is a stronger predictor of moderate to severe mental disability than SES and household income (Crosier et al. 2007), and financial hardship is strongly associated with both the onset and duration of common mental disorders (Weich and Lewis 1998). Depression (Mirowsky and Ross 2001), self-harm behaviours (Barnes et al. 2016) and increased suicide rates (Branas et al. 2015) have been linked to the experience of financial hardship. Furthermore debt, with its intrinsic links to hardship, has also been associated with a greater prevalence of substance use, depression, psychosis and suicide (Richardson et al. 2013).

Models such as the multilevel model of economic stress (Sinclair et al. 2010) acknowledge that psychological factors are implicated in the relationship between financial hardship and mental health. Research has demonstrated the importance of appraisals of financial situations: worry about debt is a better predictor of depression than amount of debt (Reading and Reynolds 2001). A number of specific psychological variables may be relevant to the relationship between financial hardship and mental health problems.

Locus of control describes the source from which an individual believes their life is determined. This may be perceived to be internal and therefore controlled by oneself, or external and at the mercy of others or from chance (Rotter 1966). An externalised locus of control has been associated with depression in low income populations (Laraia et al. 2006) and young adults exposed to economic adversity during childhood (Culpin et al. 2015). Conversely a more internalised locus of control may moderate or protect against the detrimental effects of financial stress on mental health (Krause 1987; Young 2001). Economic locus of control is the degree of control experienced over financial and occupational aspects of life and may influence the attributions about the cause of financial difficulties. Lange and Byrd (1998) found that a diminished internal locus of control was associated with increases in depression and anxiety.

Self-esteem describes a person’s sense of value and worth based on self-evaluations (Rosenberg 1965). Children have been demonstrated to show greater resilience to adverse experiences, such as poverty, when self-esteem is high (Buckner et al. 2003) and self-esteem has been found to moderate the effects of stress on life satisfaction and quality of life (Young 2001). Self-esteem may also be susceptible to the level of threat individuals attribute to their financial situation (Marjanovic et al. 2015), indicating a potential role for models incorporating perceptions of stressors. However the research into the effects of self-esteem is limited and not entirely consistent, with some research finding no vulnerability to the effects of economic strain (Waters and Muller 2003) or having no role in the protection of mental health (Ritter et al. 2000).

Hopelessness describes the sense of lacking hope and optimism regarding oneself and for the future, both in cognitions and felt sense. It can be a powerful experience, often accompanying depression and anxiety, and has been implicated as an important factor in suicide (Beck et al. 1985). Low income has been associated with increased feelings of hopelessness (Fiscella and Franks 1997). Psychological distress in welfare recipients has been attributed to feelings of hopelessness and such feelings mediate the relationship between low wages and depression (Petterson and Friel 2001). Furthermore patients reporting debt and financial concerns admitted to a psychiatric ward following a suicide attempt were found to have greater hopelessness in comparison to those not experiencing economic difficulties (Hatcher 1994). Chronically inadequate financial resources may erode hope thus, as proposed by stress process theory (Pearlin et al. 1981), increasing vulnerability to mental health problems. Alternatively, the stigma of poverty and the comparisons that individuals inevitably make to others within their society (Marmot and Wilkinson 2001) may promote hopelessness and consequently worsen mental health.

Shame is described as a painful emotion powered by the belief that one is, or is perceived by others, to be inferior or inadequate as a consequence of their thoughts, actions or behaviours, or the failure to achieve goals and expectations (Lewis 1971). Experiences of poverty, such as food insecurity, stigma and discrimination may prompt feelings of humiliation and negative self-evaluations that lead to shame. In addition people are inclined to compare themselves with others, and where personal failure against social norms is perceived, shame may follow (Marmot and Wilkinson 2001). Research on the relationship between shame, financial difficulties and mental health has been most prevalent in the study of unemployment, and has demonstrated an association with reduced mental wellbeing (Rantakeisu et al. 1999). The finances-shame model proposes that unemployment causes financial hardship and shaming experiences, the latter consequent of self and others’ perceptions of the absence of purpose and status (Starrin et al. 1997). In two samples of unemployed people financial hardship and shame significantly contributed to psychological distress (Creed and Muller 2006). The model has also been tested in the general population, providing evidence that increased financial stress, combined with a greater number of shaming experiences, reduced psychological wellbeing (Starrin et al. 2009).

A noted limitation of research on financial hardship and mental health is that most research is cross-sectional and many studies do not use standardised measures of mental health (Richardson et al. 2013). Studies also often used a single question to establish financial hardship with no assessment of reliability.

This study aims to investigate the role of four psychological factors on the relationship between financial hardship and mental health: economic locus of control, self-esteem, hopelessness and shame, using a longitudinal design and standardised measures. The following are specific hypotheses for the current study:

- Mental health difficulties will be significantly predicted by financial hardship and the psychological variables of economic locus of control, self-esteem, hopelessness and shame.
- The psychological variables of economic locus of control, self-esteem, hopelessness and shame will mediate the relationship between financial hardship and mental health difficulties
- Financial hardship will negatively affect later mental health via the mediators of economic locus of control, self-esteem, hopelessness and shame.

## Method

### Design

A longitudinal design was used with 3 month intervals between data collection.

### Participants

Participants were eligible to take part in the study if they were aged 18–65 and resident within the UK. Organisations offering support and advice to people experiencing financial difficulties, debt and receiving benefits, such as housing associations, debt support agencies, charities and food banks, were invited to assist in the recruitment of participants to the study. Participating organisations advertised the study through online platforms, using posters and leaflets within their premises, or both. The study was also advertised at student unions and those organisations with an interest in research into the relationship between money and mental health. In addition the study was advertised via social media and a website specifically designed for the purpose of recruitment. Participants also had the option of completing a paper version of the study. Participants were advised that upon participating in the study they would be entered into a prize draw. Due to the recruitment methods it is not possible to know the response rate.

Informed consent: Informed consent was obtained from all individual participants included in the study.

**Fig. 1:**
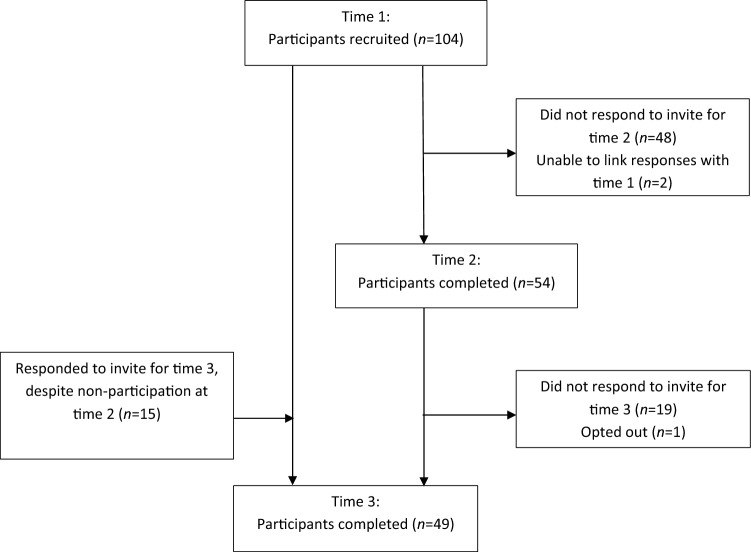
Recruitment flow diagram

At the initial data collection point the sample consisted of 104 participants, 66.3% (n = 69) completed multiple time points. Figure 1 shows the recruitment flow chart.

One participant did not complete the demographic information. Of the remainder, the average age of respondents was 40.7 years (range 19–67, SD = 12.70) and were mostly female (*n *= 78 (75%)). The ethnicity of participants was predominantly white (*n *= 94 (90.4%)). The marital status of most respondents was single (*n *= 37 (35.6%)), living with a partner (*n *= 26 (25%)) or married (*n *= 24 (23.1%)). The majority of participants had achieved some level of university education (*n *= 63 (60.5%)). Respondents reported living in private rented housing (*n *= 33 (31.7%)), social rented housing (*n *= 25 (24%)) or having a mortgage (*n *= 25 (24%)). Full or part time work was held by 43.2% (*n *= 30) of participants, and 27.9% (*n *= 29) were unable to work. A significant proportion were not working (*n *= 25 (24%)).

### Measures

- Index of Financial Stress (IFS, Siahpush and Carlin 2006): An eight item scale designed to elicit objective indicators of financial hardship in the past 6 months such as ‘went without meals’. Internal consistency in the current study at time one was acceptable at α = .74.
- InCharge Financial Distress/Financial Well-Being Scale (Prawitz et al. 2006): An eight question measure of perceived financial distress and financial well-being, Questions include ‘how often do you worry about being able to meet normal monthly living expenses?’ and ‘what do you feel is the level of your financial stress today?’. Higher scores indicate that the individual is experiencing greater financial wellbeing. Internal consistency at time one in the current study was acceptable at α = .79.
- 7-Item Generalized Anxiety Disorder Scale (Spitzer et al. 2006): A seven item questionnaire measuring symptoms of general anxiety. Participants rate on a four point scale how frequently they have experienced specific anxiety symptoms in the previous fortnight, such as ‘feeling afraid as if something awful might happen’. Higher scores indicative of greater anxiety. The scale was chosen as it is widely used in clinical and non-clinical populations. Internal consistency in the current study was excellent α = .94 at time one.
- Centre for Epidemiological Studies Depression Scale (CES-D, Radloff 1977): A 20 item scale measuring depression in the general population. Participants indicate how frequently statements such as ‘I felt hopeful about the future’ have applied in the previous week. Higher scores indicate more severe symptoms. The measure is commonly used in epidemiological studies. Internal consistency at time one in the current study was excellent α = .95.
- Perceived Stress Scale (PSS, Cohen et al. 1983): A 10 item questionnaire measuring global perceived stress. Questions include ‘in the last month, how often have you felt that you were on top of things?’. Higher scores represent greater stress. Internal consistency in the current study at time one was excellent at α = .91.
- Clinical Outcomes Routine Evaluation- General Population Version (CORE-GP) (Evans et al. 2005): A 14-item measure of general mental health/wellbeing in the general population. Participants respond how often they have felt symptoms such as ‘I have felt unhappy’ in the past week. Higher scores represent worse global mental health. Internal consistency for the current study at time one was excellent at α = .93.
- Herth Hope Index (HHI, Herth 1992): A 12 item scale measuring hope with questions such as ‘I have a positive outlook toward life’. Higher scores indicating the presence of hope. Internal consistency at time one in the current study was excellent at α = .91.
- The Other as Shamer Scale (OAS, Goss et al. 1994): An 18 item scale measuring the extent to which one feels shamed by others via questions such as ‘other people put me down a lot’. Higher scores indicate higher external shame. Internal consistency in the current study was excellent at α = .96 at time one.
- The Self-liking/Self-confidence Scale Revised (SLCS-R, Tafarodi and Swann 2001): A 16 item scale measuring self-esteem as consisting of two dimensions. Statements such as ‘I never doubt my personal worth’ indicate the extent of self-liking; and ‘I perform very well at many things’ reflects the extent of self-competence. Internal consistency at time one in the current study was excellent at α = .91.
- Economic Locus of Control Scale (Furnham 1986): A 22 item measure assessing how much control an individual perceives to have over working and financial aspects of their life. Questions include ‘there is little one can do to prevent poverty’. The scale is scored along four factors with the chronbachs alpha for each in the current sample at time one given: Internal (α = .75), Chance (α = .74), External/Denial (α = .49) and Powerful Others (α = .85). Higher scores represent less perceived control in that domain. Due to the low chronbach’s alpha for the external/denial subscale in the current sample this was not analysed in the current study.

### Procedure

Upon accessing the study online participants were provided with information about the study and a consent form. Although the option of completing paper versions of the measures was offered, no participants chose this method. Email addresses were taken as the identifying information to match responses over the course of the study and to contact for the follow-up surveys. The email address was kept separate from the answers in the data set. At approximately 3 and 6 months after the initial completion of the measures, participants were invited by email to recomplete the survey. Emails reminding participants to compete the measures were sent 1 week later. The study was conducted as part of a Doctorate in Clinical Psychology for C, ethical approval was granted from the University of Southampton Ethics Committee (Number 18791).

### Statistical Analyses

Data was analysed using SPSS V 24.0 for Windows. Missing data was substituted with the mean from the whole sample for that item. Data was assessed at all time points for adherence to assumptions of normality. Visual inspection of histograms and measures of skewness and kurtosis (within range of − 1.5 to + 1.5) of total scores at each time point, and scatterplots of all associations within and between time points, were completed for each standardised measure (full scale and subscales) to confirm both single and bivariate were normal, linear and without outliers. The suicidal ideation items, AUDIT and DUDIT did not meet assumptions of normality, therefore any analyses pertaining to these variables used non-parametric tests.

Bivariate correlations were computed to establish associations between the variables. Those factors demonstrating an association with all other variables at a significance greater than .01 in order to compensate for multiple correlations and the risk of making a type II error, were entered into hierarchical multiple regressions. Hierarchical multiple regressions were carried out using the enter method. Mental health measures were analysed separately (anxiety, depression, stress and wellbeing), with demographic and financial variables as predictors.

A priori computation of the recommended sample size to generate a moderate effect (.15), with high power (.8) for the regression analysis was calculated in G* Power (Faul 2014) as 118, in comparison with an actual sample size of 104. Assumptions of multicollinearity, homoscedasticity and independent errors were met. However collinearity was demonstrated between subjective and objective financial hardship, and self-liking and self-competence. There was no collinearity between either measure of financial hardship and the dependent variables. All the measures fell within acceptable limits for tolerance and variance inflation factors. Regression analyses was not completed with data from follow-up time points as attrition (48% at time 2 and 53% at time 3) resulted in a sample size substantially below that recommended in the G* Power computation.

A separate mediation analysis was completed for each mental health outcome (anxiety, depression, stress and wellbeing) because of the potential for variations in the mechanism by which the independent variable of financial hardship and mediators may act on the outcome. In keeping with the funnelling approach, only those variables identified as significant predictors by the regression analyses were included in a parallel multiple mediator model. PROCESS version 2.16 (Hayes 2013) was used to conduct the mediation analyses. Variables were entered into a parallel mediator model to enable the comparison of indirect effects through different mediators (Hayes 2013). Despite the reduction in sample size at time points 2 and 3, Preacher and Hayes (2008) suggest that the use of bootstrapping (5000 in the analyses for this study) permits the use of smaller samples in mediation analysis.

### Ethical Approval

The study was conducted as part of a Doctorate in Clinical Psychology for C, ethical approval was granted from the University of Southampton Ethics Committee (Number 18791).

## Results

### Correlations

Bivariate Pearson’s correlations between the standardised measures are presented in Table 1. Aside from these all other financial, psychological and mental health variables demonstrated significant correlations with one another.

**Table 1:** Bivariate correlations at time 1 (n = 104)

Variable	1	2	3	4	5	6	7	8	9	10	11	12	13
IFS	–												
PFSW	− .73**	–											
Hope	− .40**	.47**	–										
Shame	.42**	− .43**	− .75**	–									
Self-liking	− .29**	.41**	.75**	− .74**	–								
Self-competence	− .30**	.40**	.68**	− .71**	.70**	–							
LoC-internal	.21*	− .22*	− 0.12	0.02	0	0.02	–						
LoC-chance	− .39**	.43**	.35**	− .40**	.30**	.29**	− .20*	–					
LoC-powerful others	− .36**	.39**	.37**	− .43**	.40**	.36**	− .23*	.48**	–				
Anxiety	.56**	− .63**	− .64**	.64**	− .51**	− .47**	0.18	− .39**	− .29**	–			
Depression	.59**	− .65**	− .79**	.73**	− .58**	− .56**	.24*	− .41**	− .40**	.84**	–		
Stress	.54**	− .68**	− .74**	.68**	− .62**	− .58**	.21*	− .37**	− .40**	.81**	.89**	–	
Wellbeing	.57**	− .62**	− .81**	.71**	− .63**	− .59**	.21*	− .35**	− .35**	.79**	.93**	.88**	–

*IFS* index of financial stress, *PFSW* personal financial wellness scale, *LoC* locus of control

*Correlation is significant at the 0.05 level; **Correlation is significant at the 0.01 level

### Regression Analyses

Hierarchical multiple linear regressions were carried out on time one data (*n *= 104) using the enter method to see whether objective and subjective financial hardship, and the psychological variables of hope, shame, self-liking, self-competence and the locus of control subscales of internal, chance and powerful others were predictive of mental health outcomes.

Results of the regression analyses are shown in Table 2 and show that the overall model was significant for each mental health outcome. Anxiety, depression, stress and reduced wellbeing were all separately associated with decreases in subjective financial wellbeing and hope, and increases in shame. Male gender was also associated with increased depression.

**Table 2:** Linear regression final models

	Anxiety *β*	Depression *β*	Stress *β*	Wellbeing *β*
Step 1: demographics				
Age	− .04	− .03	.0	.09
Gender	.09	.12*	.04	.09
Step 2: objective FH	.11	.11	− .02	.14
Step 3: subjective FH	− .32**	− .28**	− .41***	− .22**
Step 4: psychological variables				
Hope	− .29*	− .50***	− .39***	− .52***
Shame	.37**	.34***	.24*	.27**
Self-liking	.02	.14	.0	.01
Self-competence	.08	.03	− .01	.03
LoC chance	− .05	.03	.05	.09
LoC powerful others	.13	.02	− .0	.04
LoC internal	.04	.10	.08	.05

*FH* financial hardship, *LoC* locus of control

*p < .05; **p < .01; ***p < .001

The final model significant predicted T1 Anxiety: *F*(11, 84) = 11.51, *p *< .001, *R*^2^= .60, Depression: *F*(11, 86) = 30.13, *p *< .001, *R*^2^= .79, Stress: *F*(11, 86) = 19.98, *p *< .001, *R*^2^= .72 and Wellbeing: *F*(10, 86) = 30.49, *p *< .001, *R*^2^= .77.

As the scores for the suicidal ideation items were not normally distributed, scores were dichotomised with a cut-off of 5, following the convention of previous research (Roberts and Chen 1995), splitting participants into categories approximated to ‘no ideation’ and ‘any ideation’. A logistic regression was carried out using the enter method to explore whether objective and subjective financial hardship, and the psychological variables of hope, shame, self-liking, self-competence and the locus of control subscales of chance and powerful others were predictive of suicidal ideation at time 1.

Results of the logistic regression analysis are shown in Table 3 and show that the final model was able to explain between 23.2 and 39.4% of variance in suicidal ideation at time 1. The model was found to fit the data adequately (Hosmer and Lemeshow’s *x*^*2*^ = 10.04, p = .262), and was able to predict suicidal ideation (*x*^*2*^ = 25.89, p < .01); overall the model was able to correctly predict 88.8% of all cases, though only hope successfully predicted suicidal ideation.

**Table 3:** Logistic regression final model of suicide ideation

	Cox and Snell R^2^	Nagelkerlke R^2^	Hosmer and Lemeshow x^2^	Sig	β	SE	Wald	Odds ratio exp(B)
Model	.232	.394	10.04	.262				
Predictor variable								
Age					− .02	.03	.32	.98
Gender					.44	.86	.26	1.55
Objective FH					− .14	.23	.38	.87
Subjective FH					− .02	.03	.50	.98
Hope					− .22**	.08	6.64	.81
Shame					.06	.04	2.80	1.06
Self-liking					.08	.08	.88	1.08
Self-competence					.11	.09	1.54	1.12
LoC chance					.10	.06	2.97	1.10
LoC powerful others					− .10	.07	1.84	.90

*FH* financial hardship, *LoC* locus of control

**p < .01

### Mediation Analyses

**Fig. 2:**
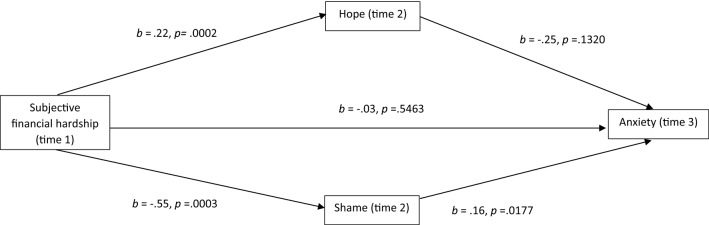
Mediational analysis of anxiety

**Fig. 3:**
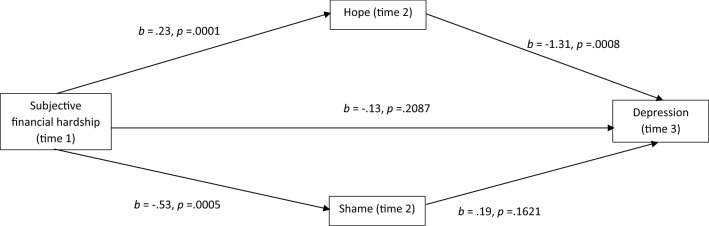
Mediational analysis of depression

**Fig. 4:**
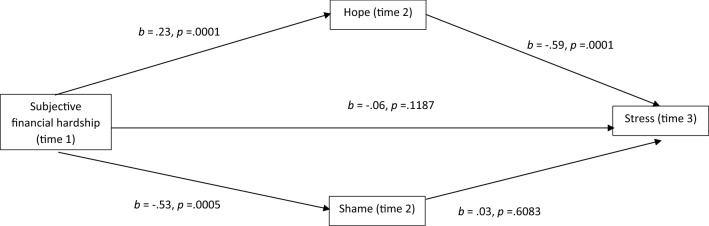
Mediational analysis of stress

**Fig. 5:**
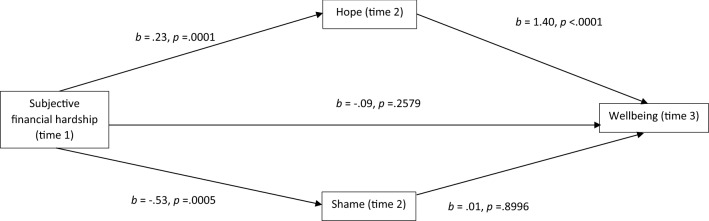
Mediational analysis of wellbeing

Hope and shame were both identified as significant predictors of anxiety, depression, wellbeing and stress in the regression analyses; while only hope was identified as a significant predictor of suicide ideation. These factors were therefore considered for their mediatory effect on the relationship between financial hardship and mental health using the longitudinal data. Table 4 demonstrates the parameter estimates for the indirect effects on the relationship between subjective financial hardship and the separate mental health outcomes, as mediated by hope and shame. Figures 2, 3, 4 and 5 show the mediation results for each mental health outcome separately.

**Table 4:** Indirect effects of subjective financial hardship on mental health through hope and shame

Mediator	*b*	SE	95% BCa CI	
			Lower	Upper
Anxiety				
Total	− .14	.05	− .24	− .07*
Hope	− .06	.04	− .14	− .01
Shame	− .09	.04	− .18	− .01*
Depression				
Total	− .40	.11	− .63	− .22*
Hope	− .30	.11	− .55	− .11*
Shame	− .10	.08	− .30	− .03
Stress				
Total	− .15	.03	− .22	− .09*
Hope	− .13	.04	− .23	− .07*
Shame	− .01	.03	− .07	− .03
Wellbeing				
Total	− .32	.08	− .52	− .20*
Hope	− .32	.09	− .54	− .17*
Shame	− .01	.06	− .14	− .11
Suicide ideation				
Total	− .08	.17	− .38	.05
Hope	− .10	.23	− .40	.08

*p < .01

Though objective financial hardship was initially significant in the hierarchical regression model, it became non-significant on inclusion of the measure of subjective financial hardship, which demonstrated a significant predictive effect of mental health outcomes in all subsequent models. Subjective financial hardship was therefore selected as the independent variable.

The effect of hope was not significant for anxiety nor suicide ideation. Figures 2, 3, 4 and 5 show that for anxiety, depression, stress and wellbeing, subjective financial hardship was positively related to hope and negatively related to shame. Thus as subjective financial wellness improved, hope increased and shame decreased. In addition hope was negatively related to depression, stress and wellbeing. Therefore higher scores on these mental health outcomes were related to reductions in hope. Shame was positively related to anxiety, thus higher anxiety scores were related to increased shame.

## Discussion

The present study hypothesized that financial hardship and the psychological variables of economic locus of control, self-esteem, hope and shame would significantly predict mental health outcomes. A hierarchal regression analyses indicated that only subjective financial hardship, hope and shame significantly predicted mental health outcomes. Objective financial hardship, self-esteem and economic locus of control did not predict mental health outcomes.

The finding that subjective financial hardship is a stronger predictor of mental health than objective financial hardship supports the work of Marjanovic et al. (2015) who found that financial threat mediated the relationship between financial situation and mental wellbeing. The importance of subjective ratings of financial difficulties is also highlighted in the multilevel model of economic stress (Sinclair et al. 2010), which positions perceptions of one’s financial situation as mediating the relationship between actual finances and mental health.

Previous research exploring the effect of self-esteem on the relationship between financial hardship and mental health has been inconsistent (Burdette et al. 2011; Hill et al. 2013; Wickrama et al. 2012). Whilst decreased self-liking and self-competence in the current study were significantly associated with increased objective and subjective financial hardship, these variables were not unique predictors of the mental health outcomes in the final regression model. The development and maintenance of self-esteem depends on a range of past and present life experiences, with financial wellness being just one of these. Self-esteem as measured in this study may therefore have been assessing a specific area of self-esteem. Self-esteem may also be dependent on the extent to which economic difficulties impact on the sense of personal agency, with reductions in the sense of control and manageability of finances reducing self-esteem to a level at which vulnerability to mental health difficulties is increased (Lange and Byrd 1998). As such the influence of self-esteem on mental health in the context of financial hardship may have a complexity beyond that analysed in the current study.

The evidence for the role of locus of control has also been inconsistent, with research demonstrating evidence both for (Krause 1987) and against (Jessop et al. 2005) an influence on the relationship between hardship and mental health. The current study specifically investigated the role of economic locus of control, finding that the internal and powerful other dimensions of economic locus of control were significantly associated with objective and subjective financial hardship, but were not unique predictors of mental health outcomes in the final regression model.

In a parallel multiple mediator model subjective financial hardship at time 1 was associated with increased shame and hopelessness at time 2. Hope at time 2 was demonstrated to have a mediatory effect on the influence of subjective financial hardship on depression, stress and wellbeing at time 3, but not anxiety. Shame at time 2 mediated the effect of subjective financial hardship at time 1 on anxiety at time 3, but not its effects on depression, stress or wellbeing. These findings partially support the hypothesis that financial hardship negatively affects mental health via the mediating variables. Stress process theory (Pearlin et al. 1981) might propose that the process of erosion of psychological factors such as hope and shame happens over an elongated time scale.

The finding of a role for shame in the development of anxiety in the context of financial hardship may reflect multiple levels of influence. On an individual level people may feel shame as a consequence of the difficulties they face in servicing the basic needs of themselves and their families, and their ability to engage in or live up to societal norms. Factors such as having to borrow money from friends and family or access benefits may increase a sense of shame. Such shaming experiences may also breed anxiety about social inadequacy through a process of internalized inferiority (Bosma et al. 2015), and some researchers propose that individuals facing financial difficulties in societies in which meritocracy is championed may be particularly vulnerable to feeling stigmatized (Bosma et al. 2012), further fuelling a sense of shame and social inadequacy.

The findings of this study add weight to the small amount of existing evidence about the role of hopelessness in the relationship between mental health and economic challenges, such as debt (Hatcher 1994) and reliance on welfare payments (Petterson and Friel 2001). The findings may be explained by stress process theory (Pearlin et al. 1981) which proposes that stressful life experiences erode psychological resources. In this case hope may protect mental health from stress by providing a sense that life stressors are temporary and amenable to resolution. In contrast the erosion of hope, and thus the presence of hopelessness, may create the sense of an interminable circumstance within which one is powerless, with deterioration in mental health as the consequence.

**Fig. 6:**
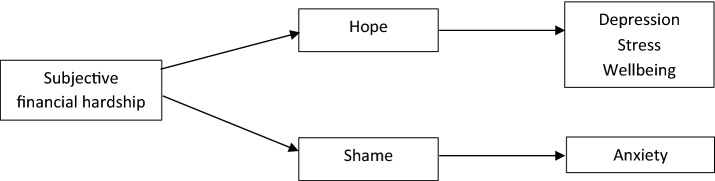
Proposed model of factors mediating the association between financial hardship and mental health

A proposed model of the mediatory influences of hope and shame is shown in Fig. 6.

### Limitations

A strength of this study is the use of standardised measures and a longitudinal design. However the time points were only over 6 months. The sample size at time 1 fell short of that recommended by G* Power (Faul 2014) by 14. This means that the findings may be underpowered, though not to a degree that impacts upon the findings. The high rate of attrition means that the mediation analysis was conducted with a small sample size. Generalisability of the findings to the general population is also problematic in light of a number of variables. Participants were disproportionally female (75%), white (90.4%), educated to university level (60.5%), and either unable to work (27.9%) or not working at all (24%). Evidence suggests that females are more likely to experience poverty (Tucker and Lowell 2015) therefore its effects may also vary by gender. Consequently the findings of the present study may not accurately reflect male experiences of financial hardship. Similarly people from black and minority ethnic groups are more likely to experience financial difficulties (Kenway and Palmer 2007). The high proportion of white people within this sample may therefore mean that the experiences of ethnic minority groups are also not represented by the findings. Given that a large proportion of the sample had achieved some level of university education, we might expect that they would also earn more, meaning their experiences of financial hardship may differ from that of the general population. That over half the sample were not working indicates there is at least some overlap between participants not working but also university educated, which questions the presumption of being higher earners. Nevertheless with such a high proportion of participants being either university educated and/or not working, the results may not represent the experiences of the general working population.

The recruitment study may mean that those with mental health problems were more likely to take part and thus the sample is not representative of the general population. Those without computer access were also likely to have been under represented in this sample.

### Clinical Implications

Increased vulnerability to mental health disorders due to hopelessness and shame resulting from financial hardship, may have long-term consequences for both individuals and communities. Funding the provision of effective and sufficient financial resource through statutory agencies may be a crucial preventative measure.

Hope has been conceptualised as dependent on a sense of agency in the face of adversity and the sense that one is able to generate solutions to difficulties. Increases in both these aspects have been shown to increase hope (Snyder et al. 1991). Specific interventions have been developed with this concept in mind, in which individuals’ barriers to hope are addressed, meaningful goals are identified and multiple possibilities for achieving those goals are generated; as well as drawing attention to and reflecting upon periods or events in which the individual has felt a sense of agency (Weis and Speridakos 2011). Such an approach could be used to generate hope in individuals. Furthermore, many therapeutic models directly address problem-solving skills in therapy, with CBT being a notable example in which a structured approach to problem-solving is taught. Wadsworth et al. (2011) found that the teaching of skills to manage poverty related stress increased emotional regulation and problem solving with positive consequences for mental health.

Therapeutic interventions that have directly targeted hopelessness in the context of suicidal ideation could also be utilized. In a CBT informed model Ghahramanlou-Holloway et al. (2014) propose that hopelessness is related to an underdevelopment in the skill of optimism and overdeveloped catastrophisation. A lack of optimism could be addressed through the development of problem-solving skills as previously discussed; while CBT is also well equipped to manage the impact of catastrophisation. The use of thought monitoring to identify triggers and responses in the context of financial hardship, and the developing of challenges to enable a person to consider these situations and thoughts from a more logical viewpoint may also be effective interventions.

The mediatory effects of shame could be targeted using Compassion Focused Therapy. Its role in increasing the functioning of soothing systems within the brain to counteract the threat systems which may be triggered by financial stressors and that breed feelings of blame and self-criticism (Gilbert 2009) may be well placed to support individuals who feel responsible either for the financial situation they find themselves in or have a sense of inadequacy in coping with the consequences. As such the development of Emotional Coping Skills, as taught within DBT (Linehan 2014), could also be utilised to support the development of the soothing systems to manage times of situational crisis and strong emotional reactions.

### Future Research

Future research should continue to attempt to address the issue of causation, using longitudinal designs of sufficient length and frequency to be sensitive to changes in psychological and mental health variables. Research looking at the way in which psychological factors interact with mental health in the context of financial strain and the mechanisms by which change occurs needs further development. The current study was conducted with a general population sample, though anyone who wished to could take part. Given that a clinical mental health population may be particularly vulnerable to challenging financial circumstances and detriment to their mental health, it will be important to explore whether and how the experience of hope and shame is impacting on their mental health. Additional research should also be conducted with groups who are at particular risk of financial hardship, such as single parents, those on low-incomes and/or receiving benefits payments and people experiencing homelessness.

## Conclusion

The results of the present study indicate that the experiences of hope and shame may mediate the relationship between financial hardship and mental health outcomes. The methodological limitations of how this study sought to measure change, and limitations in sample size and representativeness means that the conclusions that can be drawn are limited. As such, there is a need for more research to understand these relationships and add to the evidence base. In times that provide considerable financial challenges to people throughout society, understanding the means by which economic strain may increase vulnerability to mental health disorders is of great importance to facilitate the prevention of difficulties and the development of resilience.
